# Evidence-Based Application of Acupuncture in Theriogenology

**DOI:** 10.3390/vetsci9020053

**Published:** 2022-01-28

**Authors:** Gilbert Reed Holyoak, Aituan Ma

**Affiliations:** 1 Veterinary Clinical Sciences Department, College of Veterinary Medicine, Oklahoma State University, Stillwater, OK 74078, USA; 2 Department of Traditional Chinese Veterinary Medicine, College of Traditional Chinese Veterinary Medicine, Agricultural University of Hebei, Baoding 071001, China; aituan@chiu.edu

**Keywords:** acupuncture, electro-acupuncture, aquapuncture, laserpuncture, theriogenology

## Abstract

Historical evidence of acupuncture predates written history. It has been a component of Traditional Chinese veterinary medicine for many generations and is officially recognized in recorded history for treating equine disease in the Zhou Dynasty, circa 1050 BC. Drawing from a range of searchable databases, we present the use of veterinary acupuncture related to theriogenology. We touch on human-based medicine only as an introduction to current uses within veterinary medical acupuncture. This review is confined to the use of acupuncture encompassing dry needle, electroacupuncture, aquapuncture, and the few reports of laserpuncture. Starting with acupuncture’s influence on the master organs of reproduction, the hypothalamus and the pituitary glands, and the hypothalamic–pituitary–gonadal axis, we then review reports specific to the gonads—ovaries and testicles—and then its influences on the uterus. From there, we review reports on the influence of acupuncture on pain associated with reproductive surgery, and finally, on the use of acupuncture for maternal lactation. Based on published reports, we conclude that acupuncture has been shown to be effective in many situations as a treatment for infertility and/or reproductive tract disfunction, resulting in improvements in both female and male patients.

## 1. Introduction

Historical introduction to veterinary acupuncture:

Veterinary acupuncture as a component of Traditional Chinese veterinary medicine (TCVM) has been used to treat animal disease for thousands of years [1]. However, it was not until relatively recent international attention was given to its therapeutic values that its use made significant advances outside the Far East [2]. Running parallel to the increasing awareness of the anecdotal clinical value of TCVM, there has been an increasing expectation that empirical data be produced to support that which was being anecdotally reported. Over the past two decades, major strides have been made in publishing both human medicine-based, as well as veterinary medicine-based reports on the use and value of TCVM, with its acupuncture subset, in refereed scientific journals worldwide. The number of publications has blossomed over the past decade, and a comprehensive review covering the wide expanse of TCVM-related publications is not only a daunting task, but to study such a review would be equally intimidating. Therefore, the focus of this particular review will encompass refereed publications specific to the use and study of acupuncture in the field of theriogenology.

Historically, as horses played a vital role for national defense, transportation, and agriculture, acupuncture of Equidae was heavily emphasized in TCVM. Therefore, the early history of veterinary acupuncture mostly refers to equine acupuncture. The official administrative Department of Veterinary Medicine was established with full-time veterinarians to treat animal disease during the Zhou Dynasty. The classic monograph, *Zhou Li* (*The Rites of Zhou Dynasty*, published circa 1046–771 BC), recorded the administrative Department of Veterinary Medicine, indicating that veterinary medicine played a vital role during that era. Specifically, *Zhou Li* stated that all non-breeding stallions were to be castrated, and the designation of ‘equine doctor for specifically treating equine diseases’ was recorded. These doctors utilized dry needle and fire needle techniques, as well as hot iron cauterization and the surgical removal of carbuncles [3]. During the Jin Dynasty in AD 282, the *Zhen Jiu Jia Yi Jing* (*Systematic Classic of Acupuncture and Moxibustion*) first reported the use of acupuncture for infertility. It stated that the Conception Vessel (CV) channel and posterior three Yin channels were involved in the pathology of infertility [4]. Arguably, the most famous record in TCVM history, *Yuan Heng Liao Ma Ji* (*Yuan-heng’s Therapeutic Treatise of Horses*), published in 1608, mentioned that Shen-shu, Shen-peng, Shen-jiao, and Bai-hui were selected using fire needles for treating paraphimosis [5].

When reviewing the acupuncture selection rules in ancient literature for treating female infertility, Xi et al. reported that acupoints on the CV and kidney (KID) channels were often selected, and of those, CV-3, CV-4, KID-6, and KID-1 were the most frequently used. Depending on the syndrome diagnosis, these methods were often combined with moxibustion. Additionally, acupoints on the abdomen and lower limbs were commonly selected [6]. For treating semen abnormalities, Hua et al., in a review of available literature, ancient to recent, found that the ten most frequently used acupoints for treating male infertility included CV-4, Spleen (SP)-6, Bladder (BL)-23, Stomach (ST)-36, KID-3, Governing Vessel (GV)-4, CV-6, and CV-3 [7].

In this paper, we will systematically review reports on the use of veterinary acupuncture related to animal reproduction/theriogenology within the available databases. While there are multiple reports addressing the use of herbal medicine in the field of theriogenology, we will confine this review to the use of acupuncture, which will encompass dry needle, electroacupuncture, aquapuncture, and laserpuncture. We will begin with their use in influencing the master organs of reproduction, the hypothalamus and the pituitary glands, and then examine their use on the hypothalamic–pituitary–gonadal axis. From there, we will review reports specific to the gonads—ovaries and testicles—and then its influences on the uterus. We will then review reports on the influence of acupuncture on pain associated with reproductive surgery, and finally, on the use of acupuncture for maternal lactation.

## 2. Influence Directed toward the Hypothalamus and Pituitary Glands

The hypothalamic–pituitary–ovary (HPO) axis is the dominant system facilitating animal procreation by means of cyclic production of gonadotropic and steroid hormones. These processes control reproductive physiology, including the maturational responsiveness for puberty, spermatogenesis, estrous cyclicity, parturition, and postpartum endocrinological events. Electroacupuncture (EA) has been widely used to treat reproductive diseases associated with endocrinological disorders [8]. Comparing the EA stimulation of bilateral acupoints of San-yin-jiao (SP-6) and Zu-san-li (ST-36) on 36 female virgin rats to an equal number of sham-EA and controls, EA significantly upregulated gonadotrophin releasing hormone (GnRH) concentrations relative to sham and untreated controls. By 24 h following treatment with EA, serum follicle stimulating hormone (FSH) and luteinizing hormone (LH) concentrations were markedly reduced, followed by a sharp increase at day 7 and day 13. Concomitant to the fall and rise of the gonadotropin associated folliculogenesis, serum concentrations of 17β-estradiol were lower in EA-treated rats versus untreated animals at day 7, while they were higher in EA-treated rats at day 13. As expected, progesterone concentrations were lower in the EA-treated rats on both day 7 and day 13. Additionally, and importantly, serum prostaglandin E2 concentration was reduced in EA-treated rats versus untreated rats on day 1, while they were upregulated at day 7 and day 13. These results demonstrated that EA influenced the hypothalamic release of GnRH, thereby impacting the pituitary release of gonadotropins. This, in turn, affected ovarian activity and thus indicated a direct impact of the HPO axis in physiologic rats. This supports the application of veterinary acupuncture in balancing and treating reproductive diseases [9]. Through enhanced tissue repair, increased anti-inflammatory cytokine production, and analgesic relief EA is indirectly involved in treating reproductive disease. As reported in a landmark study by Salazar et al. [10], EA performed in rats and humans using limb acupuncture sites, LI-4 and LI-11, GV-14 and GV-20 (humans), and Bai-hui (rats) increased functional connectivity between the anterior hypothalamus and the amygdala and mobilized mesenchymal stem cells (MSCs) into systemic circulation.

## 3. Influence Directed on the Gonads

Progressing from the HPO axis, we now specifically focus on female gonads. In a bovine study, 57 dairy cows previously diagnosed with infertility due to inactive ovaries were randomly assigned into four groups: electroacupuncture (EA) (*n* = 15), Aqua-AP (*n* = 15), hormones (*n* = 15), and control (*n* = 12). The four acupoints used in both EA and Aqua-AP groups were Bai-hui, GV-1, and bilateral Yan-chi. In the EA group, the cows were treated for 30 min once a day for three consecutive days [11]. The Aqua-AP cows were injected with 5% dextrose at each of the four points, once daily for three consecutive days. For the hormone group, FSH was given intramuscularly twice, 48 h apart. For the control group, no treatment was given. After treatment in the EA group, 13 out of 15 dairy cows (86.7%) had a normal estrus, were inseminated, and 12 (80%) were diagnosed pregnant. In the Aqua-AP group, 9 out of 15 cows (60%) had a normal estrus and were inseminated, and 7 (46.7%) were diagnosed pregnant. In the hormone group, 12 out of 15 (80%) had a normal estrus and were inseminated, and 11 (73.3%) were diagnosed pregnant. In the control group, 4 out of 12 cows (33.3%) had a normal estrus, were inseminated, and 2 (16.7%) were diagnosed pregnant. The estrus and pregnancy rates were not significantly different between the EA and hormone groups and between the Aqua-AP and control groups, but pregnancy rates in both EA and hormone groups were significantly higher than the control and Aqua-AP groups. This study indicated that EA was an effective therapy for infertility due to inactive ovaries. Chan et al. reported a 67% pregnancy rate in chronically sub-fertile cattle, “repeat breeders”, when aquapuncture was used at Bai-hui and bilateral Shen-peng [12]. Lin, Wu, and Wu also reported the use of aquapuncture in sub-fertile dairy cattle. From a group of cattle that failed to respond to GnRH treatment and regular breeding management, a total of 18 animals (two heifers and 16 cows) that had not conceived after 3–9 attempts were treated by aquapuncture at Bai-hui and Shen-peng. The majority of the cows demonstrated estrus behavior within 14 days post-treatment and subsequently were artificially inseminated. Serum progesterone concentrations and rectal palpation were used for pregnancy diagnosis. The pregnancy rate after the treatment was 14/18 (77.7%) and 12/18 (66.6%) based on progesterone concentration and rectal palpation, respectively, with full-term pregnancy rate of 8/18 (44.4%). These results indicate that acupuncture and aquapuncture may be effective methods to treat sub-fertile, repeat breeders in dairy herds [13].

**Figure 1:**
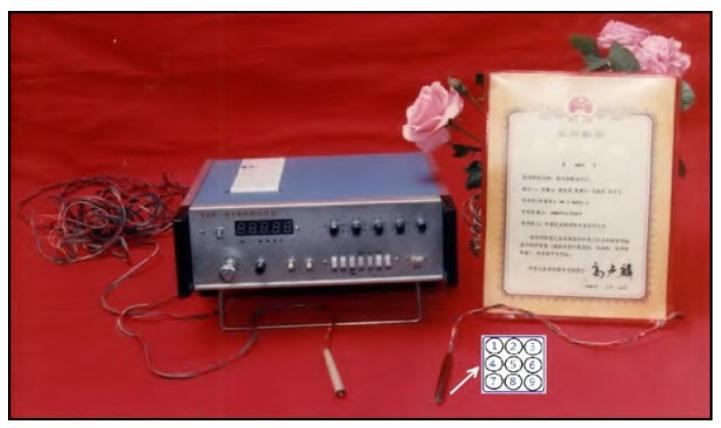
XXH-IIA Acupoint Diagnosis and Therapeutic Instrument used in this study; the white arrow points from the 1.5 cm diameter surface electrode probe to a diagram of the 9 sub-electrodes embedded in the probe head used to detect the area of lowest resistance within the acupoint; the probe on the left is the reference electrode; in this study an alligator clip electrode was used at the reference acupoint Wei-ben (used with permission).

An interesting study on the use of acupoint resistance testing followed by treatment to improve pregnancy rates in dairy cattle was reported by Luo et al. [14]. A diagnostic instrument (see Figure 1) that the authors developed, which measured electric resistance at acupuncture points, was used to determine ovarian follicular phase and optimal breeding times.

**Figure 2:**
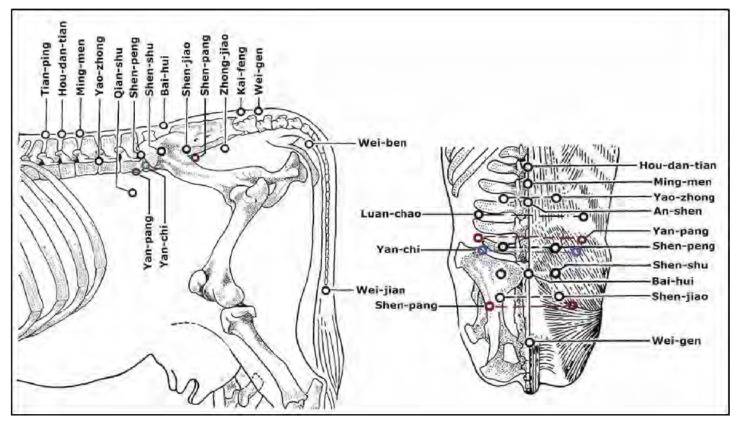
Location of acupoints Yan-pang, Shen-pang, Luan-chao, and Wei-ben used in this study and their relationship to other classical acupoints of cows. The new bovine acupoint Yan-pang (red circle) is located by identifying Bai-hui at the lumbosacral space on the midline and moving cranioventrally to the muscular depression at a line parallel to the cranial edge of the transverse processes of the 5th lumbar vertebra (left and right pictures). The other new bovine acupoint Shen-pang (red circle) is located by identifying Bai-hui at the lumbosacral space on the midline and moving caudoventrally, along the popliteal groove of the gluteus medius to the depression at the intersection of the grooves of the gluteus medius and the biceps flexor cruris (left and right pictures). The classical acupoint Luan-chao is located at the craniolateral edge of the transverse process of the 4th lumbar vertebra in line with Yan-pang (right picture). The classical acupoint Wei-ben is located on the ventral side of the tail between the 5th and 6th caudal vertebrae (left picture). (Used with permission).

Luo et al. [14] measured the resistance bilaterally at two newly described acupoints for cattle (Shen-pang and Yan-pang), which, as described, are distinct from the traditional equine points, and the classical acupoint Luan-chao (see Figure 2).

The authors did not describe the parameters for their division of the follicular phase of the estrous cycle into four separated “phases of follicular development”. However, they determined with their resistance measuring equipment a significant unbalanced resistance between left and right acupoints (*p* < 0.01) for Yan-pang during phase 2, Luan-chao during phase 3, and Shen-pang during phase 4, but not in cows with inactive ovaries. Following EA of Shen-pang and Yan-pang, a significant increase in serum 17β-estradiol concentrations (*p* < 0.05) was detected 20 min after EA in 14/25 cows (56%) and 13/42 cows (31%), respectively. EA of Shen-pang and Luan-chao produced a significant increase in serum progesterone concentrations (*p* < 0.05) in 7/20 cows (35%) and 9/13 cows (69.2%), 20 min after EA, with a trend to develop peak concentrations in 3–7 h. Unilateral EA of Shen-pang or Yan-pang (cows in phase 2 or 4, respectively) significantly increased the pregnancy rate (*p* = 0.001) in 200 cows compared to 130 controls. The authors did not report if the ovaries were palpated or if ultrasonographic examinations were performed to determine the presence or absence of a corpus luteum. Follicular wave patterns in these cows at the time of testing and time of treatment were not reported. The breeding management of the treated cows and the control cows was also not reported. However, the pregnancy rate in the treated cows indicated a significant improvement of fertility in tested and treated cows compared to similarly managed controls.

There have been several studies reported using various doses of hormones such as human chorionic gonadotropin (hCG), equine chorionic gonadotropin (eCG), and dinoprost (prostaglandin F2alpha (PGF2a)) in reduced doses to induce ovulation or luteolysis with mixed results. Ribeiro et al. reported the use of hCG in donkeys administered at Hou-hai or a sham point to induce ovulation. They compared the administration of 1500 International Units (IU) of hCG intravenously to a reduced dose of 450 IU given at Hou-hai or a sham point. They found there was no difference (*p* > 0.05) between the treatments regarding the mean diameter of the pre-ovulatory follicle, the ovulation rate, the interval between induction and ovulation, the mean diameter of the corpus luteum (CL), and serum P4 concentrations. The conclusion was that the routine dose of 1500 IU was too high for this species [15]. A study using eCG administered at Bai-hui to evaluate the effects of smaller than typically used doses of equine chorionic gonadotropin (eCG) during a fixed-time artificial insemination (FTAI) treatment program was reported by Brito et al. [16]. In three groups of dairy cows followed closely with ultrasonographic examinations and enrolled in a synchronization and FTAI program, eCG was administered to Groups 1, 2, and 3 in doses of 300 (intramuscularly (IM)), 100 (IM), and 100 (at Bai-hui) IUs, respectively. They found no significant differences between the ovulation synchronization treatment regimens for all follicular dynamic variables tested and no differences in responses to the ovulation synchronization treatment regimens for the luteal variables evaluated following ovulation. They concluded that a reduced dose of eCG administered at Bai-hui was effective in FTAI treatment regimens.

Prostaglandin F2alpha (PGF2a) is routinely used in livestock species to induce luteolysis. A study reported by Nie et al. [17] compared the conventional dose against micro doses of prostaglandin dinoprost tromethamine (PGF2a), the analogue cloprostenol, and a control of sterile water. Their aim was to assess whether administration of a micro dose of prostaglandin at Bai-hui acupuncture point offered an advantage over IM injections for luteolysis, ovulatory interval, or systemic response in mares. They used 17 mature cycling mares, wherein conventional and micro doses of dinoprost, cloprostenol, or sterile water as a negative control were administered. Treatments were assigned by dose, administration site, and treatment type (PGF2a, analogue, or sterile water). Mares were observed for ovulatory interval and systemic response to treatment. Plasma progesterone concentrations were also determined at the time of treatment and at 24 h intervals for 96 h following treatment. They found that, regardless of dose or treatment site, the ovulatory interval was shortened, and progesterone concentrations decreased in prostaglandin-treated mares, compared with control mares. No differences were observed in ovulatory intervals among prostaglandin-treated mares. However, those receiving conventional doses of PGF2alpha had greater systemic responses than mares treated with micro doses of PGF2alpha or sterile water. They concluded that administration of prostaglandin at Bai-hui was not significantly better than any other site chosen; however, a micro dose was effective at inducing luteolysis and shortening ovulatory interval regardless of administration site. The major problem with this study was the small numbers of the treatment groups, making statistical analysis almost irrelevant.

In a similar study, but conducted in cattle, Meira et al. [18] looked at the efficacy of lower doses and alternative routes of a prostaglandin F2alpha analogue, luprostiol (PGF2a), to induce luteolysis and progression to estrus in nonlactating Nelore cows (Bos taurus indicus). A conventional dose of PGF2a was compared to micro doses that ranged from 10 to 50% of the standard dose. These were administered intramuscularly (IM), intravulvosubmucosally (IVSM), or at Bai-hui. Eight days after detected estrus, cows with a corpus luteum were treated with a preselected dose of PGF2a. These researchers found that 50% of a conventional dose of PGF2a resulted in complete luteal regression (plasma progesterone < 1 ng/mL) at 48 h post-injection and hastened estrus, regardless of whether PGF was administered IM or IVSM. However, the lower doses administered at Bai-hui, or elsewhere, failed to induce complete luteal regression in nonlactating Nelore cows.

A recent study by Warsito et al. [19] using laser activation of reproduction related acupoints was reported. Using a sample of Japanese quail aged 4 weeks of age, the project was conducted for 30 days, with 4 treatments and 25 replications each for a total of 100 quails. Laser acupoint stimulation was carried out at 3-day intervals at the Ova point and 6-day intervals at Hu Men, Bei Ji, and Wei Gen points. A negative control group received no laser stimulation. A positive control group was handled as normal for laser activation; however, the laser was set at 0 Joule. T1 was a group treated with laser stimulation at a dose of 0.2 Joule, and T2 was a group treated with laser stimulation at a dose of 0.5 Joule. Their findings were that laser activation at the 0.5 Joule dose significantly increased egg quantity (egg production) and internal egg quality (Haugh unit and yolk index), without changing egg yolk color.

We now focus on male gonads. There are relatively few published studies reported in refereed journals focusing on the testicle. Those that are retrievable in our current search engines are either human or lab animal-based studies. While not veterinary acupuncture, one randomized controlled study conducted with men in Germany demonstrated that acupuncture resulted in a significantly higher percentage of motile sperm when the three motility categories were considered together (World Health Organization categories A–C) for infertile patients with severe oligoasthenozoospermia, but there was no effect on sperm concentration [20]. Using a rat asthenozoospermia model, Jin and his team demonstrated that EA bilaterally at BL-23 and ST-36 had a therapeutic effect on asthenozoospermia. Sperm viability and motility (class A, class A + B) were markedly improved following treatment once every other day for five times in the test rats. None of the EA treatments had a significant influence on sperm concentration in the asthenozoospermic rats [21]. In another study reported by Cui et al. [22] using oligozoospermic rats with insufficiency of kidney essence syndrome (OIKES), EA at BL-23 and ST-36 acupoints had significant effects on improving the sperm count, concentration, and motility. EA also markedly increased expression of vimentin and α-tubulin in Sertoli cells, significantly enhanced the immunoreactivity of proliferating cell nuclear antigen, and decreased apoptosis in germ cells. Finally, in a recent study, again by Jin et al. [23], using a rat model of idiopathic asthenozoospermia, the therapeutic effects of electro-acupuncture were reported. Transcutaneous electrical acupoint stimulation (TEAS) and electro-acupuncture (EA) at the traditional acupuncture points of BL-23, ST-36, CV-1, and CV-4 were used. The results indicated that both TEAS and EA treatments had a therapeutic effect on sperm motility and quality and could be used as a promising alternative medicine therapy for male infertility in clinical practice.

In the stallion, acupuncture treatments have not been researched or described as well as in the mare. However, there are many studies that document acupuncture for its use in pain management and muscle relaxation [24]. Reduction in inflammation, relief of myofascial pain, and improved range of motion are all benefits of acupuncture utilized commonly in performance horses [25]. While acupuncture use in stallions with back, hip and hind-limb soreness, osteoarthritis, or neurologic disease is potentially beneficial for their behavior and libido, due to the anti-inflammatory standpoint, further research is needed to elucidate the impact on gonadal and genital tract function in breeding stallions [26].

## 4. Influence Directed on the Uterus

Acupuncture has been used to increase uterine myometrial activity to improve uterine clearance. Schofield [27] reviewed a retrospective study by Rathgeber [28] involving 44 thoroughbred mares with a history of uterine fluid and/or urine pooling. These mares showed significant resolution of fluid, based on transrectal ultrasonography, on the day following the acupuncture treatment. They also had a subsequent pregnancy rate of 81% within the group. Acupuncture was initiated after traditional methods had been ineffective; however, mares were simultaneously treated with conventional methods, including oxytocin, uterine lavage, and antibiotic infusions if indicated. Therefore, acupuncture may increase fertility rates in mares with these problems that have failed to respond to conventional treatments alone. However, there was no positive control group included in this study. Rathgeber also reviewed the use of acupuncture in the treatment of uterine clearance and infertility in the mare, but no scientific research results were presented [29]. A randomized comparative study of acupuncture and exercise versus conventional ecbolic treatment of persistent post-breeding endometritis in mares was reported by Swift et al. [30]. They used 12 diagnosed mares susceptible to post breeding endometritis in a randomized crossover design using both positive and negative controls. During each estrous cycle, mares were randomized into one of six treatment groups, including stall rest, oxytocin, cloprostenol, exercise, electroacupuncture, and oxytocin with exercise. Each mare was challenged with a normal insemination dose; however, the sperm were killed beforehand. Intrauterine fluid measurements were followed to determine the associations between treatment efficacy and fluid clearance. Electro-acupuncture treatments were performed at 6h and 24h at BL-23 to BL-36 on both sides. Dry needle acupuncture was used at GB-21, Bai-hui, Yan-chi, Wei-duan, CV-1, GV-1, GV-4, and anal points. These researchers reported that compared to stall rest (negative control), exercise was the most effective treatment and had 29.7 times increased odds of fluid clearance. The second most effective treatment was oxytocin alone, with 16.9 times increased odds of fluid clearance. This was followed by cloprostenol that had 10.6 greater odds of fluid clearance, and finally, the treatment that combined exercise with oxytocin had 8.4 times greater odds of fluid clearance. Results from this study confirm that both exercise and exercise combined with oxytocin are effective methods to clear intrauterine fluid. Acupuncture under these conditions was unable to adequately assess the efficacy of uterine clearance because these mares had not previously encountered this modality. Despite application by equine veterinarians certified and experienced in acupuncture, most of these animals were resistant to the modality and became fractious. The authors suggested the need to have mares accustomed to acupuncture treatment.

In cattle, as mentioned above, aquapuncture for treatment of repeat-breeder dairy cows can improve fertility rates [13]. In a randomized controlled clinical trial in dairy cows with pyometra [31], 47 cows ultrasonographically confirmed to have purulent material within the uterine lumen, as well as the existence of a CL and a closed cervix, were assigned to one of three treatments: (1) control pyometra (CP; no treatment; *n* = 17); (2) electroacupuncture (EAP; *n* = 15); and (3) laser acupuncture (LAP; *n* = 15). Cows in the EAP group had a total of 32 acupuncture needles in sites along the Bladder and Gall Bladder meridians, bilaterally. The points specifically included were: BL-19, BL-20, BL-21, BL-23, BL-25, BL-26, BL-27, BL-28, BL-29, BL-30, BL-52, BL-54, GB-4, GB-29, GB-30, and Bai-hui. The LAP cows had cold laser therapy performed for 20 min on identical points as described above. Each cow received three acupuncture sessions on alternate days. All study cows had blood samples collected for determination of serum progesterone concentrations. Their ovaries were scanned by transrectal ultrasonography to determine the diameter of the CL. None of the study cows had serum progesterone values <1 ng/mL by the end of the monitoring period (d25), indicating the treatments did not cause luteolysis in any of the cows. After treatment completion, the study cows were maintained in pens with Holstein bulls, normally used for natural service on cows with advanced lactations. The repeated measures analysis indicated no significant differences for serum progesterone concentrations among groups. Farm cow records reviewed 150 days after treatment indicated that two cows in EAP and one cow in LAP conceived on days 38, 68, and 38, respectively, after treatment completion. In conclusion, acupuncture was not an effective treatment for persistent corpora lutea in cows with pyometra during the monitoring period. Since this study farm is a large organic certified operation, no exogenous hormones could be used. This study indicates that the acupoints and techniques used did not affect luteolysis and therefore had negligible effect on the resolution of pyometra, it being a disease of diestrus. These results suggest that a sole acupuncture approach without determining the TCVM disease pattern, with or without the integration of conventional therapies, was insufficient.

While there are multiple studies in human medicine utilizing acupuncture in uterine post-partum health, little has been reported in veterinary medicine. In a study by Korematsu et al. [32], dry needle, electroacupuncture, aquapuncture, and laserpuncture were applied to 12 traditional Japanese bovine acupuncture points (Keiketsu in Japanese) to treat delayed uterine involution. Of a group of 48 dairy cows diagnosed with delayed uterine involution, based on rectal palpation and vaginoscopic examination 21 to 35 days after parturition, 16 cows received treatment for three consecutive days. Another 32 cows were either injected intramuscularly with 25 mg PGF2a (17 cows) or received an intrauterine infusion with ampicillin (15 cows). The uterine involution following the treatment was monitored by rectal palpation and vaginoscopic examination. Milk samples were collected three times weekly and used for milk progesterone assays to monitor the ovarian function. No significant difference was observed in the uterine involution among the groups treated with moxibustion, PGF2 alpha, or ampicillin. Percentages of cows with abnormal cervical mucus and bacterial isolation from cervical swab decreased remarkably in all groups 4 weeks after treatment. Forty-six percent of cows with delayed uterine involution were diagnosed as having inactive ovaries. The percentage of cows that responded with ovulation and corpus luteum formation after moxibustion was 67 percent, slightly higher than those in cows treated with PGF2 alpha or ampicillin. Reproductive performance after the moxibustion was comparable to those after PGF2 alpha or ampicillin treatment. Results indicate that moxibustion could be used as an alternative to PGF2 alpha and antibiotics for treating delayed uterine involution in cows. Intrauterine antibiotics were the poorest choice of the three.

While the hemochorial placentation of rats is very similar to that of humans and non-human primates and a little more invasive than endotheliochorial placentation of canines, it is quite different from epitheliochorial placentation of our large domestic species. However, there are things we can glean from studies of the impact of acupuncture on the rat endometrium. In a study by Chen et al. [33], electroacupuncture was used to enhance endometrial angiogenesis in a rat model of ovarian hyperstimulation. The acupoints of SP-6 and ST-36 were chosen for electroacupuncture treatment, and the acupuncture points PC-6 and Wai-guan (TH-5) were chosen for the controls. These treatments were divided into multiple pre-treatments and peri-implantation treatments. The results support that electroacupuncture treatment can facilitate embryo implantation and improve endometrial angiogenesis during peri-implantation period. Furthermore, electroacupuncture had a superior therapeutic effect when initiated as a pretreatment. Therefore, when administered as a pretreatment, electroacupuncture may represent a simple and effective therapeutic strategy for improving the implantation rate in assisted reproduction settings. In another recent study by Xia et al. [34] the possible synergistic effect of electroacupuncture and bone mesenchymal stem cell (BMSC) transplantation was investigated in a rat model for repairing injured thin endometrium. Electroacupuncture stimulation was applied unilaterally to SP-6, CV-4, and Zi-gong (a classic point located 4 cun bilateral to CV-3). While all the damaged uteri in the treatment groups recovered to some extent, the best effects were observed in the combined BMSC and electroacupuncture group. Additionally, electro-acupuncture promoted the migration of transplanted BMSCs to damaged uteri by activating the stromal cell-derived factors. EA also improved embryo implantation rates, most likely by promoting the migration and enhancing the paracrine effect of BMSCs.

Finally, a study by the same group reported by Xi et al. [35], using the rat model of thinned endometrium, investigated electroacupuncture to improve endometrial receptivity to implantation. They tested the same acupoints as in the previous study, SP-6, CV-4, and Zi-gong, for inducing pinopodes, which are ultrastructural markers for positive embryo implantation. The results of this study indicated that the number of pinopodes in the electroacupuncture group was like that of the non-thin endometrial control group. Additionally, significantly higher expression levels of pinopode-related markers were observed in the treated group compared to the control model group. They concluded that electroacupuncture had a positive effect on the endometrial receptivity for embryo implantation.

## 5. Influence Directed on Reproductive Surgical Pain

While there are reports in the literature regarding electroacupuncture-induced analgesia sufficient for laparotomies in cattle, clinically controlled studies of the use of this modality specifically for reproductive related surgeries were not uncovered by our search engines. A recent report on the use of electro-acupuncture for anesthesia during laparotomies in goats, which they indicated was sufficient for caesarian-section surgery, was found. In 2021, Ashour et al. [36] evaluated the effect of electroacupuncture during laparotomy in 15 goats. They monitored hematological, biochemical, and physiological variables, vital parameters, cortisol hormone, pain threshold, and wound healing for the laparotomy. The ten acupoints selected for study in these laparotomy surgeries were Qi-jia (Withers) connected to ST-36 (Tsu-San-Li), Tian-ping connected to Da-Kua (Greater Trochanter), GV-20 (Bai-hui) connected to SP-6 (San-yin-jiao), BL-30 (Bai-huan-shu) connected to LIV 14 (Chi-men), and BL-30 (Bai-huan-shu) connected to TH-8 (Triple Heater). In addition, two-parallel needles pointing away from each other were inserted subcutaneously in muscle layers at the surgery site. The data (Mean ± Standard Deviation) were assessed at intervals at time 0 min before induction (control group), during induction, throughout surgery, and at 24 h after surgery for serum cortisol concentrations during and post laparotomy. The authors of this study reported that the goats showed improved rates of eyelid closure, head and neck relaxation, rumen motility, and tympany compared with the controls. There was no significant difference detected in respiratory rates, body temperatures, and capillary fill times. The total mean of hematocrit, the total mean of hemoglobin, the total mean of red blood cells, the total mean of platelets, and the total mean SPO2 were significant. Serum alanine aminotransferase (ALT) and aspartate aminotransferase (AST) showed no significance. There was significance found with eyelid closure rate, rumen motility and amount of bloating, respiratory and heart rates, and capillary refill times. Additionally, there was a significant difference in mean cortisol concentrations. Wound healing was improved by early epithelization and immature granulation tissue (at 7 days). Thick keratinized epithelization and collagen deposition in the dermal tissue with enhanced angiogenesis was seen at 14 days. There was mild restoration of skin, and the dermal tissue was well-organized at 21 days. After 28 days, there was well-formed scar tissue covering a highly cellular organized dermal tissue. They concluded that electroacupuncture “anesthesia using the 10 newly selected acupoints and two-parallel needles, at the surgery area, can produce sufficient analgesia to the flank laparotomy in goats and was superior for rumen motility and tympany suitable for goat abdomen surgery. Also, the wound healing in laparotomy goats proved excellent and better healing with electroacupuncture regime”. The main concern in reviewing this report is that concomitant intra-operative and post-op data for control goats were not clearly provided. It appears that the control parameters were assessed on the study animals just prior to induction. While the data for the study goats appeared to support the authors’ conclusions, the comparative data for goats undergoing laparotomies under local or general anesthesia were not given.

As reviewed by Xie and Sivula [37], the effectiveness of electroacupuncture for pain control in cats while undergoing ovariohysterectomy (OVH) was published by Coletto et al. [38]. Eighteen healthy cats were equally distributed in three groups: electroacupuncture, morphine, and sham controls. The cats were pre-anesthetized with acepromazine, induced with propofol, and maintained with isoflurane. Needles were introduced in sham-acupoints on animals in both the sham-control and morphine groups, and in acupoints ST-36 and GB-34 in the electroacupuncture group animals. Data was recorded just before acepromazine administration, 10 min after acepromazine administration, after anesthetic induction and stabilization, 30 min after treatment with either electroacupuncture or morphine injection, and then every 10 min for 60 min. Variables recorded were rectal temperature, respiratory frequency, heart rate, oxyhemoglobin partial saturation, mean arterial pressure, and inspired isoflurane volume. Only the animals receiving electroacupuncture did not show a significant difference between measurements for mean arterial pressure, while both electroacupuncture and morphine groups had better cardiac stability. Compared to the control group, the inspired isoflurane volume was decreased by 58.33% in the electroacupuncture group and 22.01% in the morphine group. The authors concluded that when electroacupuncture is compared to morphine, electrostimulation of ST-36 and GB-34 in cats undergoing elective OVH results in a decreased inspired isoflurane volume, leading to improved cardiorespiratory stability.

Additionally, the effectiveness of using acupuncture to relieve post-operative pain following OVH surgery in cats has been reported [39]. In this study electroacupuncture was compared to meloxicam in the management of post-surgical analgesia. Twenty-nine healthy cats were assigned to four groups: aqua-acupuncture at SP-6, GB-34, LIV-3 and ST-36 acupoints bilaterally (GA), systemic meloxicam given subcutaneously (GM), pharmacopuncture at SP-6, GB-34, LIV-3, and ST-36 acupoints bilaterally (GMFV), or a reduced dose of meloxicam given subcutaneously in the interscapular region (GMFF). Following OVH, a blinded assessor evaluated the level of discomfort at over a 24 h period using a visual analog scale and a descriptive pain scale. If the pain scale increased beyond 33% from baseline, morphine was given as an analgesic rescue. Four analgesic rescues were performed in the GM group, three in the GA and GMFF groups, and two in the GMFV group. There was no statistical difference between groups for the physiological variables, sedation, or pain scores. All treatments showed similar analgesic effects.

A similar study compared meloxicam to laserpuncture for postoperative OVH pain in the canine has recently been reported by Tomacheuski et al. [40]. In this prospective, randomized, blinded study, 16 bitches were sedated with acepromazine and randomly treated before ovariohysterectomy with systemic meloxicam or laserpuncture. For those receiving laserpuncture, stimulation was performed bilaterally at LI-4, LIV-3, SP-6, ST-36, and GB-34. Anesthesia was performed with propofol, isoflurane/O2, and fentanyl. The Glasgow Composite Measure Pain Scale (GCMPS) and Dynamic Interactive Visual Analog Scale (DIVAS) were both used to evaluate postoperative pain before and for 24 h after surgery. Morphine was administrated as rescue analgesia when pain scores were ≥3.33 (GCMPS). The results showed that dogs treated with laserpuncture presented lower GCMPS AUC for 24 h and lower GCMPS scores at 2 and 4 h postoperatively (*p* = 0.04). Three dogs treated with meloxicam required postoperatively rescue analgesia against none treated with laserpuncture. These authors concluded that, in this preliminary study, laserpuncture mitigated postoperative pain in dogs following ovariohysterectomy, and the technique is a promising adjunct to perioperative pain management in dogs undergoing soft tissue surgery.

## 6. Influence Lactation and Mastitis Treatment

In the human medicine literature, acupuncture has been well reported for treating hypogalactia/low milk production [41,42,43,44,45], and a systematic review concluded that acupuncture and acupressure are effective in increasing breastmilk volume [46]. It has also been reported for use with inflammation and mastitis [47,48,49,50].

Acupuncture has been listed as a treatment of bovine mastitis in several texts [51,52,53]. A 2013 report on dairy cattle previously diagnosed with mastitis, published by Daga et al. [54], evaluated the effects of dry needle acupuncture and aquapuncture on mastitis and milk production. The cows were divided into three groups: a dry needle acupuncture group receiving acupuncture at Bai-hui and Nyukon, a point located at the lymph node dorsal to the posterior mammary gland, approximately equal to CV-2; an aquapuncture group receiving a 1% chili pepper decoction injected at the same points; and a control group. Their study was patterned after a report by Oda et al., who used low-level laser acupuncture on subclinical mastitis [55] and, in turn, utilized a report from Russia that used electroacupuncture to treat serous mastitis in cows [56]. Daga reported that the cows receiving acupuncture had the highest milk production over the three months observation period following treatment; however, the level of mastitis did not decrease in that group, although it also did not increase as much as in the control group. The cases of mastitis decreased in the aquapuncture group, with milk production being higher than in the controls. The group sizes were only six cows each, so the statistical power was severely limited in this study [54].

Rayan, Klopfenstein, and Kutzler [57] have published a more recent report on the use of acupuncture as an adjunctive complementary treatment in subclinical bovine mastitis as diagnosed by an elevated somatic cell count in their milk. Ten cows were treated with dry needle acupuncture compared to nine control cows. For front quarters affected by subclinical mastitis, the acupuncture points used were SP-12, SP-17, SP-18, SP-21, ST-18, and CV-12. For rear quarters affected by subclinical mastitis, the acupuncture points used were BL-30, BL-30-1, BL-49, KID-10, CV-2, and CV-3. These authors concluded that while acupuncture did not affect the somatic cell count, milk ion conductivity, bacteriology results, or lactate dehydrogenase activity, it did reduce N-acetyl-beta-D-glucosaminidase (NAGase) activity in cows with subclinical mastitis. This reduction indicates that acupuncture may promote healing of the damaged mammary epithelial cells, which are the primary source of NAGase activity in milk serum. While this is encouraging, acupuncture studies without the use of intramammary antibiotics and studies comparing the effects of using different acupuncture points for the treatment of subclinical mastitis in dairy cattle are needed.

## 7. Discussion and Conclusions

In conclusion, the influence of acupuncture on the major components of reproductive physiology, from basic endocrinology of the hypothalamic–pituitary–gonadal axis, gonadal function, to the tubular organs of the reproductive tract and mammary gland function has been reviewed herein (see Table 1). Acupuncture modalities, from dry-needle, electroacupuncture, aquapuncture, to laserpuncture, have been shown to be effective in many situations as a treatment for infertility and/or disfunction, resulting in improvement in both female and male patients. Acupuncture can be considered a successful treatment in restoring a level of fertility in patients by improving sperm quality, ovarian function, and balancing the reproductive endocrine system. To date, there was no reported adverse event involving acupuncture for treating infertility. Although the studies reviewed herein indicate a positive influence of veterinary acupuncture employing various modalities used for the reported reproduction associated conditions, many of the studies were on limited numbers of animals, thereby affecting statistical power. Therefore, due to the small number of published studies, inadequacy of procedures and/or insufficient information for analysis, and high levels of heterogeneity, further large, well-designed random controlled and blinded trials are required for a more definitive conclusion as to the effectiveness of these acupuncture modalities for treating reproductive tract conditions.

## Figures and Tables

**Table 1:** Overview of acupuncture points and modalities utilized directed at each major component of the reproductive tract.

Major Component	Acupoints Used	Modality Used
Hypothalamus and pituitary	SP-6; ST-36; LI-4; LI-11; GV-14; Bai-hui	EA
Gonads: Ovary Testicle	GV-1; Yan-chi; Shen-peng; Shen-pang; Yan-pang; Luan-chao; Hou-hai; Bai-hui	EA Aqua
Gonads: Testicle	BL-23; ST-36; CV-1; CV-4;	EA
Uterus	BL-19; BL-20; BL-21; BL-23; BL-25; BL-26; BL-27; BL-28; BL-29; BL-30; BL-36; BL-52; BL-54; GB-4; GB-21; GB-30; SP-6; ST-36; CV-1; GV-1; GV-4; Bai-hui; Yan-chi; Wai-guan; Wei-duan; Zi-gong	EA DN Aqua Laser
Reproductive Surgery	Qi-jia; ST-36; Tian-ping; Da-Kua; GV-20 (Bai-hui); SP-6; LIV-6; LIV-14; BL-30; TH-8; GB-34	EA Aqua
Mastitis	Bai-hui; Nyukon/CV-2; SP-12; SP-17; SP-18; SP-21; ST-18; CV-12; BL-30; BL-30-1; BL-49; KID-10; CV-3	Aqua DN

SP: Spleen; ST: Stomach; LI: Large Intestine; GV: Governing Vessel; EA: Electroacupuncture; BL: Bladder; CV: Conception Vessel; GB: Gall Bladder; DN: Dry Needle; LIV: Liver; TH: Triple Heater; KID: kidney.

## Data Availability

Not applicable.
